# Pro-inflammatory T cells-derived cytokines enhance the maturation of the human fetal intestinal epithelial barrier

**DOI:** 10.1016/j.isci.2024.109909

**Published:** 2024-05-07

**Authors:** Francesca P. Giugliano, Marit Navis, Sarah Ouahoud, Tânia Martins Garcia, Irini A.M. Kreulen, Evelina Ferrantelli, Sander Meisner, Jacqueline L.M. Vermeulen, Manon van Roest, Jean-Noël Billaud, Jan Koster, Yousif Dawood, Bernadette S. de Bakker, Daisy I. Picavet-Havik, Irene M. Schimmel, Nicole N. van der Wel, Pim J. Koelink, Manon E. Wildenberg, Joep P.M. Derikx, Wouter J. de Jonge, Ingrid B. Renes, Ruurd M. van Elburg, Vanesa Muncan

**Affiliations:** 1 Department of Gastroenterology and Hepatology, Tytgat Institute for Liver and Intestinal Research, Amsterdam Gastroenterology Endocrinology Metabolism, Amsterdam University Medical Center (AUMC), University of Amsterdam, Amsterdam, the Netherlands; 2 QIAGEN Digital Insights, 1001 Marshall Street, Redwood City, CA, USA; 3 DNAnexus, 204 El Camino Real, Mountain View, CA, USA; 4 Center for Experimental and Molecular Medicine (CEMM), Amsterdam University Medical Center (AUMC), University of Amsterdam, Amsterdam, the Netherlands; 5 Department of Obstetrics and Gynecology, Amsterdam University Medical Center (AUMC), University of Amsterdam, Amsterdam, the Netherlands; 6 Amsterdam Reproduction and Development research institute, Amsterdam University Medical Center (AUMC), University of Amsterdam, Amsterdam, the Netherlands; 7 Department of Medical Biology, Electron Microscopy Center Amsterdam, Amsterdam University Medical Center (AUMC), University of Amsterdam, Amsterdam, the Netherlands; 8 Department of Pediatric Surgery, Pediatric Surgery Center of Amsterdam, Amsterdam University Medical Center (AUMC), University of Amsterdam, Amsterdam, the Netherlands; 9 Department of Pediatrics, Amsterdam University Medical Center (AUMC), Emma Children’s Hospital, Amsterdam, the Netherlands; 10 Danone Nutricia Research, Utrecht, the Netherlands

**Keywords:** Immunology, Cell biology, Developmental biology

## Abstract

Small intestine (SI) maturation during early life is pivotal in preventing the onset of gut diseases. In this study we interrogated the milestones of SI development by gene expression profiling and ingenuity pathway analyses. We identified a set of cytokines as main regulators of changes observed across different developmental stages. Upon cytokines stimulation, with IFNγ as the most contributing factor, human fetal organoids (HFOs) increase brush border gene expression and enzyme activity as well as *trans*-epithelial electrical resistance. Electron microscopy revealed developed brush border and loss of fetal cell characteristics in HFOs upon cytokine stimulation. We identified T cells as major source of IFNγ production in the fetal SI lamina propria. Co-culture of HFOs with T cells recapitulated the major effects of cytokine stimulation. Our findings underline pro-inflammatory cytokines derived from T cells as pivotal factors inducing functional SI maturation *in vivo* and capable of modulating the barrier maturation of HFOs *in vitro*.

## Highlights

• HFOs retain stabile expression of gut maturation markers *in vitro*
• Cytokine mix, particularly IFNγ, induces brush border maturation in HFOs
• IFNγ boosts both epithelial barrier and brush border maturation *in vitro*
• IECs-CD3^+^cells co-culture induces brush border development and barrier function

## Graphical abstract

**Figure:**
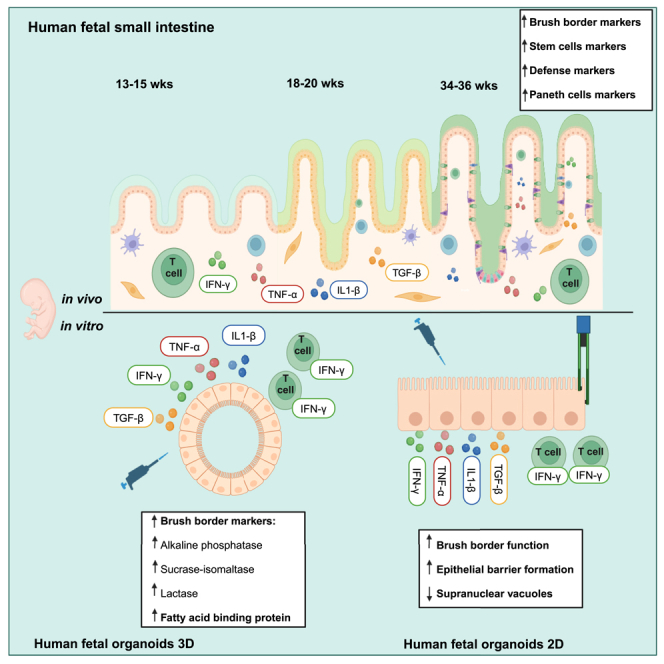

## Introduction

The importance of the first 1,000 days of life (from conception till the child’s second year of life) on setting the fundaments of human health has become increasingly evident in recent years. During this time, the course of gastrointestinal (GI) tract maturation can influence the susceptibility to disease in neonatal and adult life.^1^^,^^2^ A healthy GI tract performs various functions such as: effective absorption and digestion of nutrients, optimal barrier integrity, and a home to stable and resilient microbiome.^3^ The GI tube is formed *in utero* around the 6–8^th^ week of gestational age and while the morphological gut development is completed by the 22^nd^ week of gestation, maturation of the gut functions will proceed after birth, during the first two years of life.^4^^,^^5^^,^^6^^,^^7^ The maturation of digestive functions is aligned with developmental stage and diet.^8^ Before birth the fetus is fed via the umbilical cord and this type of feeding needs to secure the development and growth of the whole organism. The expression of brush border enzymes such as lactase (*LCT*) and sucrase-isomaltase (*SIS*) starts *in utero* and increases gradually after birth. The expression of *LCT* peaks at birth with introduction of milk diet while *SIS* continues to increase its expression even after 6 months of age in context of digesting carbohydrates derived from solid food. The gradual increase of expression by the intestinal epithelial cells (IECs) of these epithelial brush border enzymes necessary for nutrient digestion is also a part of barrier maturation.^8^ Moreover, infants are born with a relative leaky gut allowing passive transport of macromolecules from the mother’s milk into and across IECs.^9^ Another hallmark of neonatal IECs is a highly developed apical canalicular system (ACS) that feeds luminal macromolecules from milk into large intracellular vacuoles. This facilitates the transport of such molecules across the epithelial barrier without loss of their biological activity. However, during the first postnatal months in mice, and after 22 weeks of gestation in the human fetal proximal small intestine (SI), the intestinal barrier matures leading to a more selective barrier and intracellular vacuoles cease to exist.^5^^,^^10^^,^^11^ In addition to providing a physical barrier, the intestine exerts several other defense functions, including the secretion of anti-microbial peptides, and modulation of the mucosal immune system responding to stimuli and regulating homeostasis.^12^ Mucin and anti-microbial peptide secretion can be detected *in utero* but increases significantly after birth.^13^ In addition, the mucosal immune system is functionally naive at birth and shows a different composition and activity compared to the adult mucosal immune system.^14^^,^^15^ Gut maturation is a genetically intrinsic process to the IECs that can be modulated by external factors particularly hormones and cytokines as well as environmental stimuli, like microbial signals and dietary factors.^16^^,^^17^^,^^18^^,^^19^^,^^20^^,^^21^^,^^22^ Any delay or changes in this process may lead to a misbalance, and therefore to the onset of pathologic conditions either in early life such as malabsorption, infection, growth retardation, inflammation and sepsis, or in both childhood and adulthood such as increased susceptibility to inflammatory bowel disease (IBD), neurodevelopmental impairment, and allergies.^23^^,^^24^^,^^25^^,^^26^^,^^27^ To support the optimal intestinal development, to ensure intestinal health, and prevent disease during early and later life, it is imperative to gain more detailed knowledge on the mechanisms of the intestinal barrier maturation. The limiting factor for studying human intestinal barrier maturation is the lack of human tissue specimens from late fetal and neonatal stages of life, due to obvious ethical constrains, and insufficiency of thoroughly characterized neonatal *in vitro* models. With the development of the intestinal organoids (IOs) system,^28^ it is possible to mimic different aspect of the gut epithelial functions *in vitro*. Moreover, the possibility to generate small intestinal human fetal organoids (HFOs) opened the possibility to investigate some of the aspects of the human immature intestine.^29^ Previously, our group demonstrated that IOs derived from late stages of mouse GI development recapitulate the main physiological events that characterize the transition from the neonate to adult life, underlying the intrinsic component of suckling-to-weaning transition of IECs.^30^ Although useful in studying the gut maturation, the murine *in vitro* models cannot be fully extrapolated to the human situation due to species differences in the timing of GI maturation. In mice, several maturation changes occur only after birth while for humans they already take place *in utero.*^4^ In this study, we aimed to carefully assess the potential of HFOs for studying intestinal maturation. We first performed gene expression analysis of fetal intestinal tissue samples aged between 13 and 20 postconceptional weeks together with preterm neonatal tissue to identify *in vivo* markers of human intestinal maturation and clarify the milestones of SI functional development. We next investigated the expression of these markers across multiple passages of 17–20 weeks HFOs culture to establish the intrinsic potential of HFOs to mature *in vitro*. Next, by performing *in silico* analysis we identified a set of pro-inflammatory cytokines as potential upstream regulators of the GI developmental changes *in vivo*. Finally, we showed that upon cytokine stimulation, HFOs lose their immature characteristics underlying the potential of pro-inflammatory cytokines as signal to induce the functional maturation of the intestinal barrier.

## Results

### Global gene expression profile of human fetal intestinal tissue reveals a set of potential maturation markers

**Figure 1:**
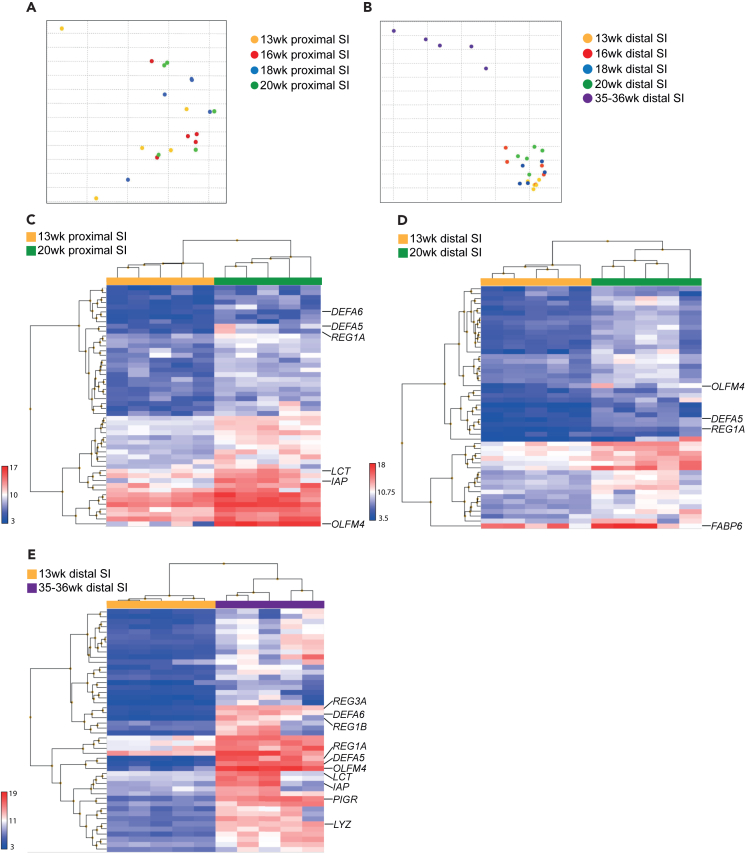
Gene expression analysis revealed the set of developmental gut maturation markers composed of genes involved in absorption, defense, and stem cell function Principal component analysis (PCA) was conducted on global gene expression profiles of human fetal (A) proximal and fetal and neonatal (B) distal small intestinal tissue, gestational age 13, 16, 18, 20, and 35–36 weeks. Hierarchical clustering of the top 50 upregulated genes in (C) 20 weeks proximal tissue compared with 13 weeks proximal tissue, (D) 20 weeks distal tissue compared to 13 weeks distal tissue and (E) 35–36 weeks distal tissue compared with 13 weeks distal tissue. Specific genes chosen as being most upregulated and of biological interest are highlighted. *n* = 5 independent donors per group. Colored scale bar represents normalized gene expression values per sample. See also Figure S1, Table S1.

To identify the differentially expressed genes that could serve as potential maturation markers of the human intestine, we first compared human intestinal tissue at different developmental stages. Since intestinal maturation can differ along the proximal-to-distal GI axis,^31^ we separately analyzed proximal and distal gut segments. For both the proximal and distal intestine we investigated gestational stages of 13, 16, 18, and 20 weeks by gene expression analysis. Additionally, for the distal gut tissue, 35–36 weeks neonatal tissue obtained from surgical interventions was also included. Principal component analysis (PCA) revealed limited separation of tissue samples by gestational age for proximal tissue (Figure 1A). For distal tissue, the separation by gestational age was more substantial, and mainly driven by separation between the fetal tissues versus neonatal tissue (Figure 1B). We next aimed to identify key maturation markers *in vivo*. Differential expression analysis revealed genes that were significantly upregulated in the tissue of late compared to early gestational age, and clustering of samples according to gestational age (Figures 1C–1E, and Table S1). A subset of maturation markers for further evaluation was selected according to the level of upregulation, known epithelial expression and biological interest. In 20 weeks proximal tissue, there was a significant upregulation of stem cell marker olfactomedin 4 (*OLFM4*) compared to 13 weeks proximal tissue (Figure 1C, and Table S1). In addition, the brush border enzymes intestinal alkaline phosphatase (*IAP*) and *LCT* were higher expressed, together with several defense markers (*DEFA5*, *DEFA6*, and *REG1A*) (Figure 1C, and Table S1). In the distal SI, the fatty acid binding protein 6 (*FABP6*) was higher expressed in 20 weeks compared with 13 weeks tissue (Figure 1D, and Table S1). Comparison between 35–36 weeks and 13 weeks distal tissue revealed an upregulation of the same genes for stem cells *OLFM4,* brush border enzymes and absorption (*IAP* and *LCT*) and for defense activity (*DEFA5*, *DEFA6*, *REG1A*, *REG1B*, and *REG3A*) (Figure 1E). Additional defense markers increased at this neonatal stage compared to fetal tissue were polymeric immunoglobulin receptor (*PIGR*) and lysozyme (*LYZ*) (Figure 1E, and Table S1).

The set of maturation markers of interest was subsequently confirmed in the tissue samples by RT-qPCR. Consistently with the gene expression analysis, in neonatal versus fetal stage, we found a significant increase of *OLFM4*, *IAP*, *LCT*, *DEFA5*, *REG1A*, *LYZ*, and PIGR expression with *OLFM4* and *IAP* being higher also between 20 and 13 weeks gestational age in proximal SI (Figure S1). In summary, we identified a set of gut maturation markers specific to proximal and distal intestinal segments and related to developmental specific gut functions.

### Majority of maturation markers do not increase over time during prolonged organoid cultures suggesting persistence of fetal phenotype *in vitro*

**Figure 2:**
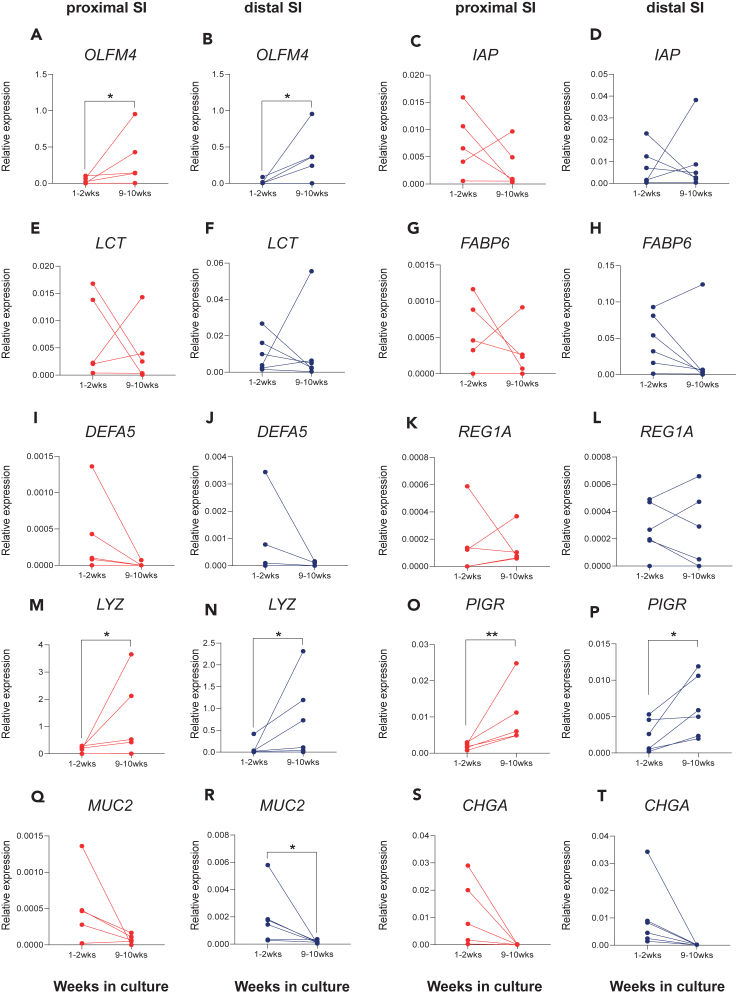
Prolonged human fetal organoid cultures retain fetal features in terms of brush border, absorption, defense, goblet and enteroendocrine cells markers expression when cultured for up to 10 weeks (A–T) RT-qPCR analyses in proximal (red) and distal (blue) HFOs cultures at 1–2 weeks of culture and 9–10 weeks of culture of (A and B) *OLFM4*, (C and D) *IAP*, (E and F) *LCT*, (G and H) *FABP6*, (I and J) *DEFA5*, (K and L) *REG1A*, (M and N) *LYZ*, (O and P) *PIGR*, (Q and R) *MUC2*, and (S and T) *CHGA*. *n* = 5 proximal and *n* = 6 distal independent donors, data are plotted as paired values of individual cultures. ∗*p* < 0.05, ∗∗*p* < 0.01, as determined by a ratio-paired t test.

Previously we have shown that the late stages of the mouse fetal intestinal cells possess an intrinsic potential to undergo transition to adult intestinal cells during prolonged organoids culture.^30^ Besides, some studies reported maturation of human fetal cells *in vitro*^32^ while others claimed human fetal cells to be stable overtime.^33^ To determine whether human fetal intestinal epithelium has the potential to mature *in vitro*, we monitored the expression of the identified maturation markers across multiple passages HFOs cultures. Primary epithelial cells were isolated from human fetal intestinal tissue (17–20 weeks gestational age) and maintained in culture approximately for 9–10 weeks. We compared expression levels at the start of the culture (i.e., 1–2 weeks of culture) with expression levels after long term culture (i.e., 9–10 weeks of culture) to determine if the maturation markers expression increases over time. Independent HFOs cultures originating from different donors showed a significant increase in *OLFM4* expression in both proximal (Figure 2A) and distal SI (Figure 2B) after 10 weeks of culture. The expression of the brush border enzymes *IAP* (Figures 2C and 2D), *LCT* (Figures 2E and 2F) and enterocyte marker *FABP6* (Figures 2G and 2H) was variable between individual organoid cultures, resulting in no significant changes in both proximal and distal cultures. Among the defense markers there was a decrease of *DEFA5* (Figures 2I and 2J) overtime, while *REG1A* (Figures 2K–2L) stayed unchanged in both proximal and distal cultures. Defense markers *LYZ* and *PIGR* showed a significant increase at 9–10 weeks compared to the start in both intestinal segments (Figures 2M–2P, respectively). Additionally, we investigated the expression of other IEC type markers such as the mucin 2 (*MUC2*) for goblet cells and chromogranin A (*CHGA*) for enteroendocrine cells since the number of these cells is also known to increase prior to birth. However, the expression of these markers in HFOs long term cultures was reduced (Figures 2Q–2T, respectively). All together, these findings demonstrate that 17–20 weeks HFOs do not undergo an intrinsic maturation *in vitro* and retain a relatively stable fetal phenotype over multiple passages in culture.

### Inflammatory cytokines drive intestinal development changes *in vivo* and increase brush border markers expression of 3D HFOs *in vitro*

**Figure 3:**
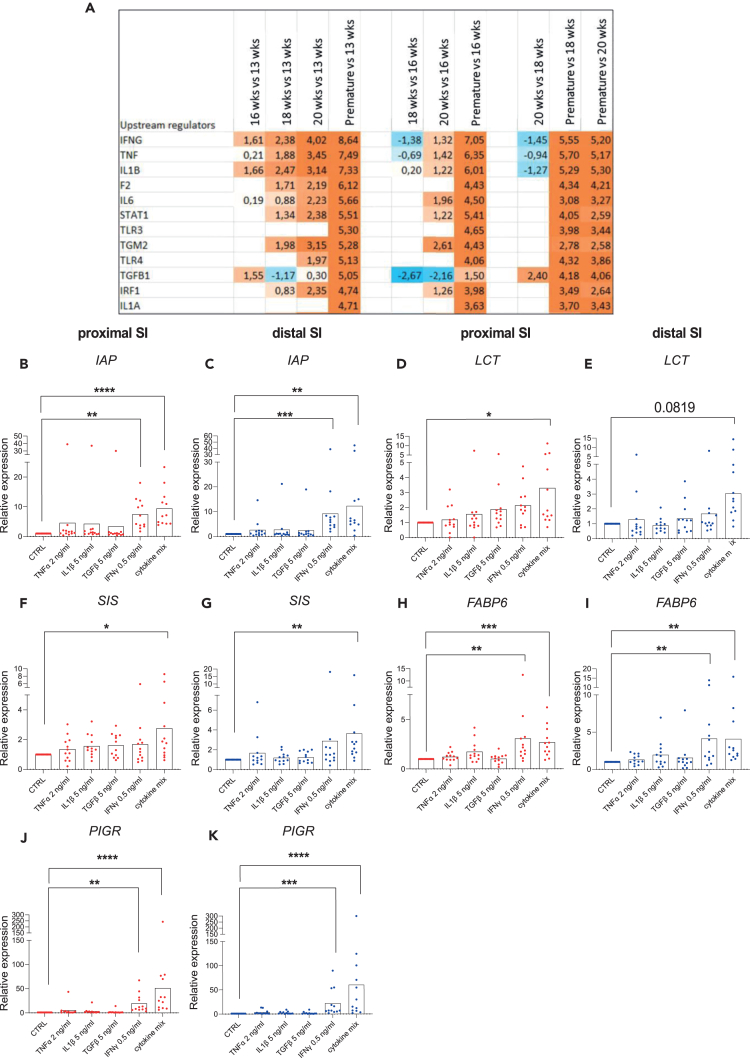
Inflammatory cytokines are upstream regulators of gene expression changes between neonatal and fetal stages and induce increased expression of brush border, absorption, and mucosal defense markers *in vitro* (A) Ingenuity pathway analysis showed inflammatory cytokines IFNγ, TNFα, IL1β, and TGFβ1 as upstream regulators of intestinal changes between late and early gestational age tissue and between neonatal versus fetal tissue. Activation *Z* score is presented by number and color. Orange color represents activation status of upstream regulator while blue signifies inhibition. (B–K) RT-qPCR analyses of maturation markers in proximal (red) and distal (blue) small intestine HFOs of (B and C) *IAP*, (D and E) *LCT*, (F and G) *SIS*, (H and I) *FABP6* and (J and K) *PIGR*. *n* = 12 independent donors, data are plotted as paired values of individual cultures. ∗*p* < 0.05, ∗∗*p* < 0.01, ∗∗∗*p* < 0.001, ∗∗∗∗*p* < 0.0001 as determined by non-parametric one-way ANOVA. See also Figures S2–S4.

**Figure 4:**
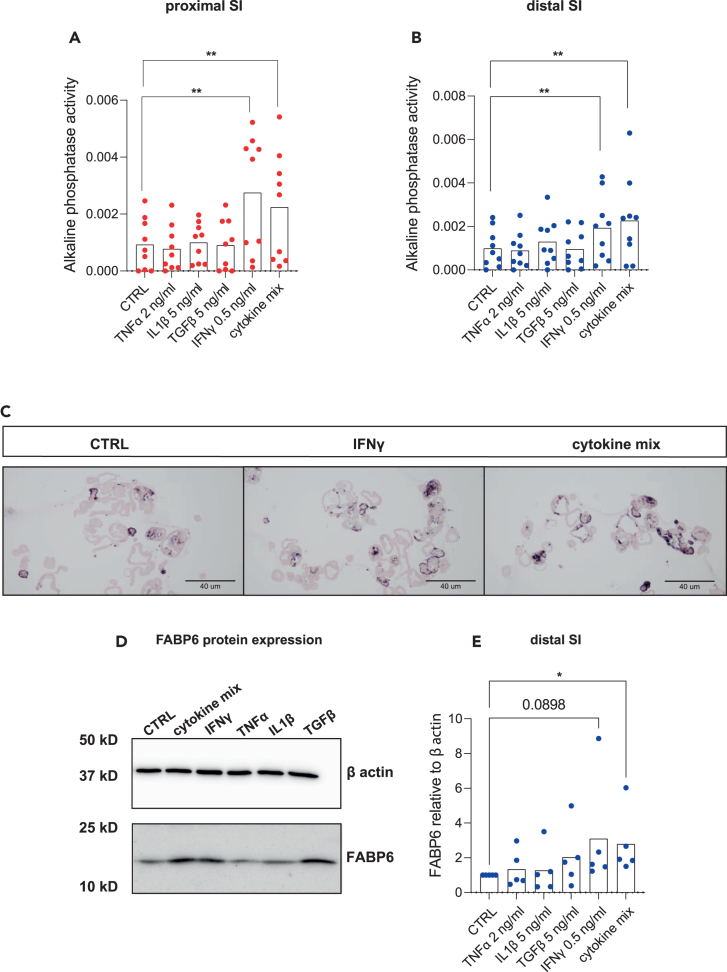
IFNγ and cytokine mix stimulation increases IAP activity and FABP6 protein expression in HFOs (A and B) Alkaline phosphatase activity level (μg pNPP/min) in (A) proximal and (B) distal HFOs upon cytokines stimulation (*n* = 9 independent donors). (C) Alkaline phosphatase (IAP) staining of embedded HFOs upon IFNγ, and cytokine mix stimulation compared to control. (D) FABP6 protein expression measured by western blot in distal HFOs. (E) FABP6 western blot quantification on *n* = 5 independent donors. ∗*p* < 0.05, ∗∗*p* < 0.01 as determined by non-parametric one-way ANOVA.

To identify tissue factors capable of inducing the intestinal epithelial maturation we performed upstream regulator analysis by ingenuity pathway analysis software (IPA, QUIAGEN Digital Insights).^34^ The IPA identified TNFα, IL1β, TGFβ, and IFNγ as the main upstream regulators of the gene expression changes between late and early gestational stages as well as between neonatal and late gestational tissue (Figure 3A). Following IPA prediction, we assessed the potential of these cytokines to induce the functional SI epithelial maturation *in vitro*. We first performed titration experiments to determine the optimal concentration of each cytokine capable of inducing downstream targets without causing cell damage (Figures S2A–S2F). Next, we stimulated HFOs cultures for 72 h with either each cytokine or the cytokine mix. We observed a significant gene expression increase of brush border enzymes *IAP*, *LCT*, and *SIS* (Figures 3B–3G) upon cytokine mix stimulation compared to the control as well as of the enterocyte marker *FABP6* (Figures 3H and 3I) and the defense marker *PIGR* (Figures 3J and 3K) in both proximal and distal SI HFOs cultures. The expression of stem cell markers *OLFM4* (Figures S3A and S3B) and *LGR5* (Figures S3C and S3D) decreased upon IFNγ and cytokine mix stimulation in both intestinal segments. This is in line with the notion that both IFNγ and cytokine mix stimulation induced more differentiation. The expression of Paneth cells defense marker *LYZ* (Figures S3E and S3F) remained unchanged, while the expression of *MUC2* and *CHGA* was only increased in the proximal SI (Figures S3G–S3J). Critically none of the maturation markers was changed in organoid cultures generated from neonatal or adult tissues (Figure S4). As the changes in the gene expression upon cytokines stimulation of fetal organoids were the most obvious for the epithelial brush border gene expression, we further investigated these findings by performing functional assays in HFOs 3D cultures. Stimulation with both the cytokine mix and IFNγ alone significantly increased IAP enzyme activity in both proximal and distal SI (Figures 4A and 4B). Consistently, IAP showed a more intense protein staining on stimulated organoids (Figure 4C) compared to control. The same result was observed for FABP6 protein expression, as assessed by western blot in the distal SI (Figures 4D and 4E). Overall, our data show enhanced expression and function of brush border markers in HFOs cultures upon cytokine stimulation, in particular IFNγ.

### Cytokine mix and IFNγ contribute to the development of the 2D intestinal epithelial barrier and maturation of the epithelial brush border

**Figure 5:**
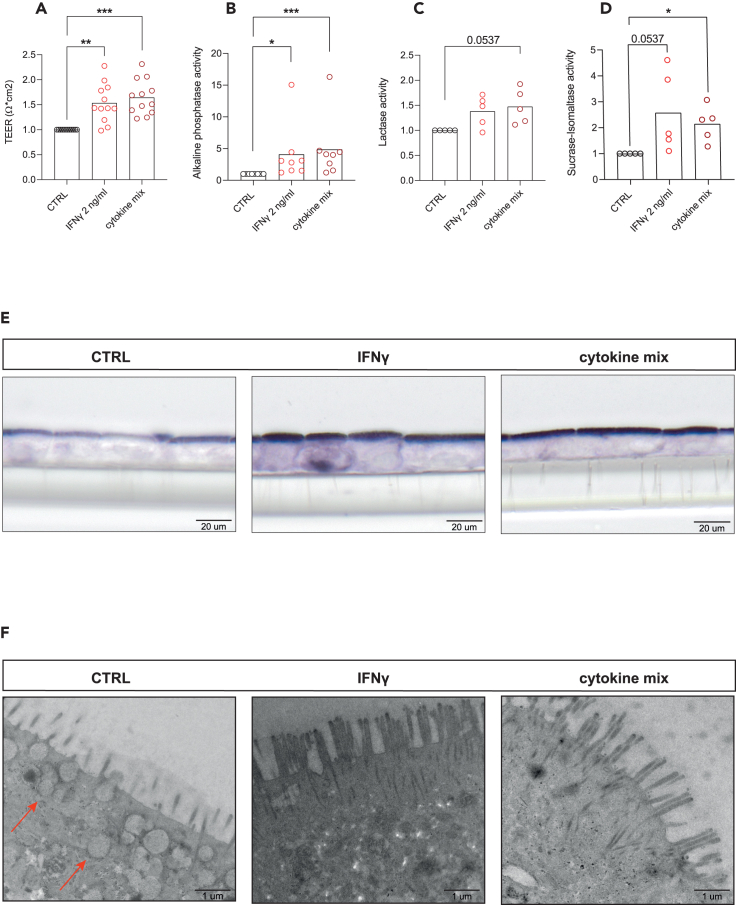
IFNγ and cytokine mix stimulation enhance epithelial barrier formation, brush border enzyme activity and disappearance of supranuclear vacuoles in the 2D intestinal monolayer (A) TEER values for organoids monolayers upon IFNγ and cytokine mix stimulation compared to control (*n* = 12 independent donors). (B–D) (B) Alkaline phosphatase (μg pNPP/min, *n* = 8 independent donors), (C) LCT (μg glucose/min converted, *n* = 5 independent donors), and (D) SIS activity levels (μg glucose/min converted, *n* = 5 independent donors), measured on organoids 2D monolayers upon IFNγ and cytokine mix stimulation compared to control. (E) Alkaline phosphatase staining showing 2D monolayer in embedded transwell upon IFNγ and cytokine mix stimulation compared to control. (F) Transmission electron microscopy (TEM) shows loss of vacuoles (red arrows) upon IFNγ, and cytokine mix stimulation compared to control. ∗*p* < 0.05, ∗∗*p* < 0.01, ∗∗∗*p* < 0.001, as determined by non-parametric one-way ANOVA.

To further assess the function of the epithelial barrier, we generated 2D monolayers from single fetal IECs on transwell inserts and measured transepithelial electrical resistance (TEER). We measured higher TEER upon basolateral stimulation with cytokine mix and in particular IFNγ, compared to the control condition (Figure 5A). In addition, the enzymatic activity of IAP, LCT, and SIS was increased upon IFNγ and cytokine mix stimulation (Figures 5B–5D). Consistently with 3D culture condition, IAP protein expression was higher upon IFNγ and cytokine mix stimulation in 2D monolayer (Figure 5E). To assess the structural changes imposed by inflammatory cytokines we next performed transmission electron microscopy (TEM) that revealed improved microvilli organization upon IFNγ stimulation compared to the control condition (Figure 5F). Interestingly, we also found a decrease of supranuclear vacuoles in the IFNγ and cytokine mix condition compared to the control (Figure 5F). Supranuclear vacuoles are structures that are part of the ACS in suckling mammals and are linked to an immature barrier.^10^ They function as the absorption reservoirs of macronutrients from milk,^35^ hence their reduction can be regarded as a sign of epithelial maturation. Together, these data suggest that both cytokine mix and IFNγ increases intestinal barrier maturation *in vitro* by increasing the barrier integrity and brush border functions.

### T cells produce IFNγ in the fetal intestine and promote the maturation of fetal intestinal epithelial cell barrier

**Figure 6:**
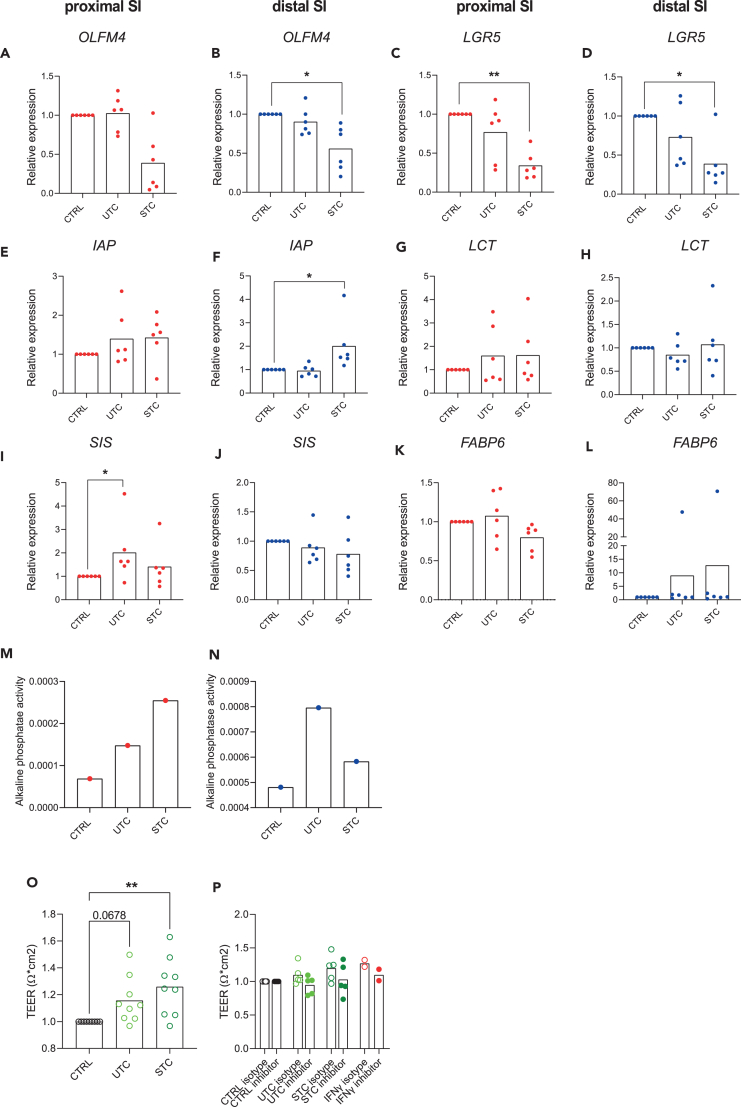
Co-culture of T cells and 3D/2D epithelial cells increases the expression of brush border and absorption markers and epithelial barrier formation. This effect is partially lost upon IFNγ inhibition (A–L) RT-qPCR analyses in proximal (red) and distal (blue) SI HFOs of (A and B) *OLFM4*, (C and D) *LGR5*, (E and F) *IAP*, (G and H) *LCT*, (I and J) *SIS*, and (K and L) *FABP6* gene expression upon HFOs 3D/T cells co-culture, *n* = 6 independent donors, data are plotted as paired values of individual cultures. (M and N) Alkaline phosphatase activity levels (μg pNPP/min) in proximal (M) and distal (N) small intestine upon HFOs 3D/T cells co-culture (*n* = 1 donor). (O) TEER values for organoids monolayers upon 2D/T cells co-culture (*n* = 9 independent donors). (P) TEER values for organoids monolayers upon 2D/T cells co-culture with or without the presence of IFNγ inhibitor (*n* = 5 independent donors). ∗*p* < 0.05, ∗∗*p* < 0.01, as determined by non-parametric one-way ANOVA. See also Figure S5.

We next attempted to identify the source of inflammatory cytokines, in particular of IFNγ, as a major mediator of the cytokine mix effect used in our experiments. Therefore, we used of the publicly available RNA sequencing (RNA-seq) dataset of intestinal fetal cells at 18 postconceptional weeks^36^ and identified T cells as a major source of IFNγ production (Figure S5A). Subsequently, we isolated T cells from the lamina propria of fetal intestines (Figure S5B) to perform co-culture with both 3D and 2D organoids. Since we were unsure whether T cells *in vitro* could produce cytokines in the absence of *in vivo* stimuli we included in our experiments two different T cells conditions: unstimulated T cells (UTC) and stimulated T cells (STC). Both UTC and STC were able to produce IFNγ, although levels produced by STC were higher than that produced by UTC (Figure S5C). Gene expression levels of *OLFM4* and *LGR5* were decreased upon HFOs co-culture with STC, (Figures 6A–6D) similar to what we observed upon 3D stimulation with the human recombinant IFNγ and cytokine mix conditions. After 72 h of co-culture, HFOs showed a trend to increased gene expression levels of the brush border gene IAP (Figures 6E and 6F) in both proximal and distal SI HFOs. We also observed increased expression of *LCT,* although not significant (Figure 6G), and increased *SIS* (Figure 6I) in proximal SI HFOs and a not significant increase of *FABP6* in both proximal and distal SI HFOs (Figures 6K and 6L). IAP activity level was higher in both UTC and STC co-cultures in the proximal (Figure 6M) and in the distal small intestinal HFOs (Figure 6N). Importantly, 2D/STC co-culture significantly increased TEER values of the intestinal monolayer (Figure 6O). This effect was partially lost when IFNγ activity was inhibited (Figures 6P and S5D). These data demonstrate the capability of T cells derived cytokines, and particularly IFNγ, to enhance the maturation of the epithelial brush border barrier in early life as described before.

## Discussion

The initial aim of our study was to define the markers of human intestinal maturation *in vivo* and assess the potential of HFOs organoid culture as a model system to study human gut maturation *in vitro*. Previous studies that assess the capability of HFOs to mature *in vitro* are limited and discordant. For example, it has been shown that fetal organoids undergo methylation changes *in vitro* that are associated with some degree of maturation,^32^ while another study showed a stable gene expression of a set of human maturation markers along multiple culture passages.^33^ We performed microarray analysis of multiple fetal and neonatal stages to identify suitable maturation markers with increment changes between early and late fetal and neonatal stages. As expected, and partially reported previously,^37^ the main human gut maturation changes in the epithelium were associated with mechanisms of nutrients digestion and microbial defense. The choice of the maturation markers for further investigation was based on the level of upregulation during fetal stages and fetal versus neonatal stages as well as biological and functional significance.^30^ The separation of proximal and distal intestinal segments was done due to their different functions regarding digestion, nutrient absorption, and defense.^38^ Aligned with this strategy, we separately evaluated HFOs cultures from proximal and distal intestinal segments for maturation potential *in vitro*. We showed that these cultures have a limited potential to mature after prolonged passages that is restricted only to the stem cells marker *OLFM4* and the defense markers *LYZ* and *PIGR*. *OLFM4* was previously identified as a human intestinal maturation marker^33^^,^^37^ and prolonged HFOs cultures showed a consistent increased expression of *OLFM4* over time.^37^ Likewise, the expression of *LYZ*, which is reported to be expressd by Paneth cells in SI around 20 weeks of gestation,^2^^,^^39^ was increased during prolonged HFOs cultures. Another defense marker that was upregulated in prolonged HFOs cultures is *PIGR*. This Fc receptor is responsible for the transcytosis of secretory IgA (sIgA), helping protecting the mucosal epithelium from pathogens.^40^ Likely, expression of these markers is at least partially intrinsically regulated by epithelial cells. On the other hand, *DEFA5* and *REG1A* expression is lost during culture suggesting that these genes require extra stimuli to increase or maintain their expression such as microbial signals.^41^ The expression of brush border markers *IAP*, *LCT*, and *FABP6* significantly increases in later stages of fetal life and at birth compared to the 2^nd^ trimester of pregnancy.^42^^,^^43^ In our microarray data, we showed that the expression of these markers gradually increases form early to late fetal and neonatal stages. *In vitro* expression of *IAP*, *LCT*, and *FABP6* remained unchanged during the long-term HFOs cultures suggesting that the epithelial barrier requires additional signals to complete the maturation process *in vitro* and does not recapitulate the same maturation pattern observed *in vivo*. These data are in line with Senger et al., suggesting that HFOs retain their specific gestational program *in vitro*.^33^ Therefore, the human maturation program that would reinforce fetal organoids to transit to the adult state would require extra epithelial signals.^44^ The IPA showed that a set of pro-inflammatory cytokines was among the upstream regulators driving the major gene expression changes between the tissue of different fetal and neonatal stages. We hypothesized that these could be the crucial extrinsic factors contributing to the IECs functional maturation during gut epithelial development. In support of our hypothesis, the stimulation of HFOs with the mix of the four cytokines induced a significant increase in the expression of brush border markers *IAP*, *LCT*, and *SIS*. *SIS* is another disaccharidase required for the processing of sucrose therefore important from weaning onwards.^33^ Moreover, the cytokine mix increased the expression of *FABP6* and the defense marker *PIGR*. Even though *PIGR* was intrinsically upregulated during prolonged HFOs cultures, its increased expression upon cytokines stimulation is in line with previous works showing that the expression of this gene is regulated by IFNγ and other cytokines leading to NF-Κb pathway activation.^45^^,^^46^ Indeed, among the cytokines used in our study, IFNγ seemed to have the major contributing effect in the upregulation of *IAP*, *FABP6*, and *PIGR*. Although used at a low dose, it is striking to observe that cytokine mix and IFNγ alone induced maturation of the epithelial cells in terms of brush border enzymes expression and barrier formation, rather than damaging of the intestinal epithelial barrier as it is described in specific pathologic conditions such as IBD.^47^ Additionally, upon IFNγ and cytokine mix stimulation, there is a clear reduction of supranuclear vacuoles on the apical side of the enterocytes that can be interpreted as a sign of barrier maturity.^10^^,^^48^ In our study, we showed that the main intestinal source of IFNγ during the fetal stage are T cells. Intestinal immune cells milieu starts its development around 2^nd^ trimester of gestation and its abundance increases throughout gestation.^49^ It has been shown that cytokine production starts during the fetal stage as the fetal intestinal tissue resident memory (TRM) T cells, responsible for mucosal homeostasis and infections response, actively produce TNFα and IFNγ as well as other cytokines.^49^^,^^50^ Schreurs et al. showed that stimulated fetal intestinal CD4+T cells produce TNFα, IL-2, and IFNγ and that TNFα has a supportive role in HFOs growth and proliferation *in vitro.*^50^ Our results support the interaction between developing immune and epithelial compartments during intestinal development as co-culture between CD3^+^ IFNγ producing cells and IECs induces the expression of intestinal maturation markers in HFOs and increases barrier formation in fetal intestinal 2D monolayers. These findings emphasize the important role of the immune system, which actively contributes to the maturation of the gut epithelial barrier during the fetal development by the production of cytokines which at low doses are not harmful but rather supportive for the IECs development. In line with our findings, hPSC-derived IOs retain fetal phenotype in culture^37^ but when co-cultured with Jurkat T cells, the maturation of major epithelial defense and absorption functions is induced via the IL-2/STAT3 pathway.^51^ In our work we focus mainly on the role of IFNγ that was the most significantly predicted factor among the top upstream regulators extrapolated by IPA. In an elegant mice study, Nabhani et al. showed that after birth the gut microbiota induces the so called “weaning reaction” when an increase of IFNγ and TNFα occurs, during a specific time frame, to favor the normal development of the immune system and to prevent the onset of inflammatory disease later in life.^52^ A study by Hill et al. shows that 24 h after *E. coli* microinjection in hPSC derived organoids, genes related to NF-kB signaling and cytokine production were upregulated. Interestingly, gene set enrichment analysis at 48 h and 96 h post-microinjection showed an increased gene expression related to innate anti-microbial defense, epithelial barrier production, and tissue maturation hallmarks.^53^ Based on this and our data it can be speculated that the activation of inflammatory-related pathways in epithelial cells, seems important also after birth to positively guide the intestinal epithelial changes toward the final mature stage. In summary, this study demonstrates that HFOs are limited in their capacity to recapitulate the intestinal fetal maturation *in vitro* and that maturation of the intestinal barrier can be modulated by immune related factors. In this context, we were able to show that T cells-derived inflammatory cytokines, and in particular IFNγ, at low levels, are able to activate epithelial signaling, that might serve during early life as support for the epithelial barrier development and function under homeostatic conditions. Our study demonstrates that human intestinal barrier maturation benefits from a crosstalk with T cells derived signals that starts already prior to birth to modulate and support proper barrier function, which can be relevant for the development of new therapeutic strategies for prevention and treatment of intestinal diseases related to prematurity.

### Limitation of the study

This study has few limitations. The first is absence of proximal SI tissue from neonates, due to unavailability of intestinal tissue samples at this stage of life. Therefore, the maturation markers for proximal SI could not be interrogated by global expression analyses. Another limitation of this study is the exclusion of factors from other cell types residing in the intestinal mucosa, apart from T cells, that could contribute or enhance epithelial barrier maturation in early life.

## STAR★Methods

### Key resources table

**Table:** 

REAGENT or RESOURCE	SOURCE	IDENTIFIER
**Antibodies**		

IFNγ antibody inhibitor	R&D	Cat# MAB285; RRID: AB_2123306
IgG2B Isotype Control	R&D	Cat# MAB004; RRID: AB_357346

**Biological samples**		

Human fetal tissue	AUMC	This manuscript
Human neonatal tissue	AUMC	This manuscript
Human adult tissue	AUMC	This manuscript

**Chemicals, peptides, and recombinant proteins**		

Advanced Dulbecco’s Modified Medium (DMEM/F12)	Invitrogen	Cat# 12634-028
Glutamax 100x	Invitrogen	Cat# 35050-038
Penicillin-Streptomycin	Invitrogen	Cat# 15140-122
Hepes 1M	Life Technologies	Cat# 15630-056
N2 supplement 100x	Invitrogen	Cat# 17502-048
B27 supplement 100x	Invitrogen	Cat# 17504-044
n-Acetylcysteine	Sigma-Aldrich	Cat# A9165-5G
Epidermal Growth Factor	Invitrogen	Cat# PMG8045
[Leu15]-gastrin	Sigma-Aldrich	Cat# G9145
Nicotinamid	Sigma-Aldrich	Cat# N0363
A83-01	Tocris	Cat# 2939
SB202190	Sigma-Aldrich	Cat# S7067
mNoggin conditioned medium	AUMC	In-house made
Rspondin1 conditioned medium	AUMC	In-house made
Wnt3a conditioned medium	AUMC	In-house made
Human Intesticult (HI) medium	Stemcell Technologies	Cat# 06010
Iscove’s Modified Dulbecco’s Medium (IMDM)	Invitrogen	Cat#2 1980-065
Heat inactivated fetal bovine serum (FBS)	Sigma/Aldrich	Cat# F7524
Paraformaldeyde	Sigma/Aldrich	Cat#1.04003
HBSS	Westburg/Capricon	Cat# HBSS-3A
Matrigel, phenol red-free	Corning®	Cat# 356231
TrypLE express	Invitrogen	Cat# 12605036
Cell recovery solution	Corning®	Cat# 354253
RHO/ROCK pathway inhibitor (Y)	Sigma	Cat# Y0503-5MG
Collagen type I	Ibidi	Cat# 50201
HistoGel	Thermo Scientific	Cat# HG-4000-012
Acetci acid	Merk/Millipore	Cat# 1000631000
Collagenase IV	Sigma	Cat#C5138-500MG
Percoll	VWR of Fisher Sc.	Cat# 17-0891-01
1,4- Dithiothreitol (DTT)	Sigma	Cat# D9779-5G
Ethylenediaminetetraacetic acid (EDTA)	Invitrogen	Cat# 15575-038
2-Mercaptoethanol	Sigma	Cat# M3148-100ML
Cell lysis buffer	Cell signalling/bioke	Cat# 9803S
recombinant human (rh) IFNγ	R&D systems	Cat# 285-IF
recombinant human (rh) TNFα	Peprotech	Cat# 300-01A
recombinant human (rh) IL1β 5	Thermo Fisher Scientific	Cat# PHC0814
recombinant human (rh) TGFβ	Peprotech	Cat# 100-21A
Sucrose	Sigma-Aldrich	Cat# 84097-250G
Lactose	Sigma-Aldrich	Cat# L3625-1KG
Maleic buffer	Merk/Millipore	Cat# 800380
Peroxidase/glucose oxidase	Sigma/Aldrich	Cat# P7119-10CAP
3,3′-Dimethoxybenzidine dihydrochloride 8mM	Sigma/Aldrich	Cat# 191248-25G
Tris–HCl 0.5 M	Sigma/Aldrich	Cat# T3253
Phosphatase substrate (pNPP)	ThermoFisher Scientific	Cat# 34045
Nitro blue tetrazolium chloride/5-bromo-4-chloro-3-indolyl-phosphate	Roche	Cat# 1681451001
Phorbol 12-myristate 13- acetate (PMA)	Santa Cruz/Bio-Connect	Cat# sc-3576A
Ionomycin (IONO)	Invitrogen	Cat# I-24222
MicroBeads	Miltenyi Biotec	Cat# 130-050-101
MS columns	Miltenyi Biotec	Cat# 130-042-201
TRI-reagent	Sigma-Aldrich	Cat# T9424-200ML
Bioline ISOLATE II RNA Mini kit	Bioline	Cat# BIO-52073
Revertaid reverse transcriptase	Thermo Fisher Scientific	Cat# EP0442
Annexin V	BD Pharmingen BD	Cat# 556419
Propidium Iodide	Sigma/Aldrich	Cat# P4170
SYBER No-ROX Kit	GC-biotech	Cat# Bio-98020

**Critical commercial assays**		

Click-iT® plus EdU Alexa Fluor® 647 Flow Cytometry Assay Kit	Invitrogen	Cat# C10634
IFNγ DuoSet ELISA	Bio-Techne	Cat# DY285B-05

**Deposited data**		

Raw and analyzed transcriptomic data	This manuscript	Geo repository: GSE247523
Raw scRNA-seq data (human fetal duodenum)	Holloway et al., 2021^36^	ArrayExpress: E-MTAB-9489

**Oligonucleotides**		

36b4 forward: TCATCAACGGTACAAACGA reverse: GCCTTGACCTTTTCAGCAAG	In-house designed	This manuscript
βACT forward: AGAGCTACGAGCTGCCTGAC reverse: AGCACTGTGTTGGCGTACAG	In-house designed	This manuscript
CYCLO forward: CACCGTGTTCTTCGACATTG reverse: TTCTGCTGTCTTTGGGACCT	In-house designed	This manuscript
EEF1A1 forward: ACATCCACACACTGTTGAAGGA reverse: ATGTTGCCTGATGCCTGGATA	In-house designed	This manuscript
GAPDH forward: AAGGTGAAGGTCGGAGTCAA reverse: AATGAAGGGGTCATTGATGG	In-house designed	This manuscript
H2AFZ forward: CCTCACCGCAGAGGTACTTG reverse: GTTGCAAGTGACGAGGGGTA	In-house designed	This manuscript
HPRT forward: AGTTCTGTGGCCATCTGCTT reverse: GTTAAACAACAATCCGCCCA	In-house designed	This manuscript
PGK1 forward: CCACTGTGGCTTCTGGCATA reverse: ATGAGAGCTTTGGTTCCCCG	In-house designed	This manuscript
POLR2A forward: CGGAGATTGTCACCCCCTTC reverse: CCATCACACATGTGCCGTTC	In-house designed	This manuscript
RPL32 forward: TGACAACAGGGTTCGTAGAAG reverse: GCGGTTCTTGGAGGAAACATTG	In-house designed	This manuscript
RPL4 forward: TGGCCAGGGTGCTTTTGGAA reverse: AGCAAAACAGCTTCCTTGGTCT	In-house designed	This manuscript
TBP forward: TCTCATGTACCCTTGCCTCC reverse: GTGCACAAATAATGCCCCTT	In-house designed	This manuscript
IAP forward: TCAGCTGGGTACTCAGGGTC reverse: ATCGCCACTCAGCTCATCTC	In-house designed	This manuscript
CHGA forward: TGACCTCAACGATGCATTTC reverse: CTGTCCTGGCTCTTCTGCTC	In-house designed	This manuscript
DEFA5 forward: AGTCTGGGGAAGACAACCAG reverse: GGACTCACGGGTAGCACAAC	In-house designed	This manuscript
FABP6 forward: GGCAAGTTCGAGATGGAGAG reverse: TTGCTTTCCTTGCCAACAGT	In-house designed	This manuscript
IRAK1 forward: CTCAGCGACTGGACATCCTT reverse: AGTGTGCTCTGGGGTGCTTCT	In-house designed	This manuscript
IRF1 forward: TGGCTGGGACATCAACAAGG reverse: GCGACAGTGCTGGAGTCA	In-house designed	This manuscript
LCT forward: GGCAGTCTGGGAGTTTTAGG reverse: ATGCCAAAATGAGGGCAAGTC	In-house designed	This manuscript
LGR5 forward: AATCCCCTGCCCAGTCTC reverse: CCCTTGGGAATGTATGTCAGA	In-house designed	This manuscript
LYZ forward: GCTGGAGACAGAAGCACTGA reverse: GTGGATCACGGACAACCCTC	In-house designed	This manuscript
MUC2 forward: TGTAGGCATCGCTCTTCTCA reverse: GAGTCCATCCTGCTGACCAT	In-house designed	This manuscript
NFKBia forward: AAAGCCAGGTCTCCCTTCAC reverse: CAGCAGCTCACCGAGGAC	In-house designed	This manuscript
OLFM4 forward: CTCCATGATGTCAATTCGGA reverse: CAGAGTGGAACGCTTGGAAT	In-house designed	This manuscript
PAI forward: TTGGTGAAGGGTCTGCTGTG reverse: GTGCTGCCGTCTGATTTGTG	In-house designed	This manuscript
PIGR forward: CCCTGAAGTCAGATCAACGG reverse: AGCACGAAGAGCAGCATTG	In-house designed	This manuscript
REG1A forward: CCGTGAGACCTGGGTTGATG reverse: GGATTAACACTGCTTGGGGC	In-house designed	This manuscript
SIS forward: TTTCCAGTTGTCTTTTGCAATG reverse: CAATTTGCATGGAAGCTGAG	In-house designed	This manuscript

**Software and algorithms**		

FACSDiva	BD Bioscience	Version 9.2
FlowJo	Treestar	Version 10.3
ImageJ	National Institutes of Health	Version 1.52a
CFX Maestro Software	BioRad	Version 5.3.022.1030
LinRegPCR	Amsterdam, The Netherlands	Version 2015.4
Olympus microscope	CellSense Entry	Version 4.1

### Resource availability

#### Lead contact

Further information and requests for resources and reagents should be directed to and will be fulfilled by the lead contact, Vanesa Muncan (v.muncan@amsterdamumc.nl).

#### Materials availability

Human organoids have been generated and used for experiments at the Tytgat Institute for Liver and Intestinal Research. Due to data anonymity, it was not possible to identify Donor ancestry, race or ethnicity and gender due to Donor privacy laws. The unavailability of these information did not influence our analysis.

#### Data and code availability

• Transcriptomic data have been deposited at gene expression omnibus (GEO) and are publicly available as of the date of publication. Accession numbers are listed in the key resources table.
• This paper analyzes existing, publicly available data. These accession numbers for the datasets are listed in the key resources table.
• All data reported in this paper will be shared by the lead contact upon request.
• The paper does not report original code.
• Any additional information required to re-analyze the data reported in this paper is available from the lead contact upon request.

### Experimental model and study participant details

#### Human donors

Human fetal intestinal tissue samples derived from fetal abortions (gestational age 13-20 weeks) were obtained by the HIS Mouse Facility of the Academic Medical Center of Amsterdam (AUMC), the Netherlands. All Material has been collected from donors from whom a written informed consent for the use of the material for research purposes had been obtained. These informed consents are kept together with the medical record of the donor by the clinic.

Human fetal tissue was also obtained from the Dutch Fetal Biobank (DFB), located at the AUMC, after a written informed consent was signed prior to delivery. Ethical approval for the DFB was granted by the Medical Research Ethics Committee AUMC (protocol number 2016_285, #B2017369).

Human neonatal ileum tissue samples were obtained at intestinal surgery at the Pediatric Surgery Center of the AUMC. Neonates underwent surgery for necrotizing enterocolitis (NEC) between 35-36 weeks of postmenstrual age. Tissue samples were obtained with the approval of the ethical committee of the AUMC (protocol number W16_161 #16.188). Written informed consent by the infants’ guardians and adult patients for the use of the material for research purposes was obtained. Samples included were non-inflamed by visual inspection and histological evaluation. Adult intestinal tissue was collected in accordance with the Biobank Review Committee of the AUMC, after all patients had given informed consent (protocol number 178#A201470). All methods were performed in accordance with the relevant guidelines and regulations, as stated in the AUMC Research Code, in a certified laboratory (ISO15189 accreditation M304).

### Method details

#### Human fetal organoids (HFOs) generation

HFOs were isolated from fetal small intestine derived from human fetal abortions, according to an established protocol.^54^ After the identification of the appendix, the proximal and the distal parts of the small intestine were cut open, reduced to small pieces, and washed extensively in PBS. Subsequently, tissue was incubated for 1 hour (h) in ethylenediaminetetraacetic acid (EDTA) 5mM (Invitrogen, cat#15575-038)/ DL-Dithiothreitol 2mM (Sigma - cat#D9779-5G) at 4°C on a shaker (400–600 rpm). After the incubation, crypts were mechanically detached from the tissue and the supernatant was filtered through a 70 μm strainer and centrifuged at 950 rpm for 10 minutes (mins) at 4°C. Subsequently, pellets were washed in cold advanced DMEM/F12 1:1 (Invitrogen, cat#12634028) supplemented with 100 U-mg/mL penicillin/streptomycin (Invitrogen, cat#15140122), 10 mM HEPES (Life Technologies, cat#15630-056) and 1x Glutamax (Invitrogen/gibco, cat#35050-038) and plated in Matrigel (Corning, cat#356231) in a 24 wells plate and placed at 37°C for 15 mins before adding HISC growth medium (produced by Tytgat Institute, key resources table). HFOs cultures were maintained in HISC medium at 37°C, 5% CO2. HISC medium was refreshed every 4 days and cultures were passaged by mechanical disruption weekly.

#### Neonatal and adult organoids generation

Neonatal or adult tissue specimens were sectioned, open longitudinally and subsequently rinsed in ice-cold PBS. Following this, the mucosa was separated from the submucosa, and the mucosal tissue was then cut into approximately 5 mm fragments. These mucosal fragments were incubated for 1 hour at 4°C in a solution of 0.5 M EDTA /1 M DTT in DMEM-F12. After the incubation, the isolation of organoids from the mucosal tissue was performed as described for the human fetal tissue.

#### RNA isolation and gene expression analysis

Human fetal intestinal tissue was snap frozen and stored at -80°C until processed. RNA was isolated from the tissue with TRI-reagent (Sigma-Aldrich, cat#T9424-200ML) and purified with the Bioline ISOLATE II RNA Mini kit (Bioline, cat#BIO-52073). To obtain RNA from HFOs, one well was collected in lysis buffer with 2-mercaptoethanol 1:100 (Sigma, cat#M3148-100ML) and stored at -80°C until isolation. RNA was isolated using the Bioline ISOLATE II RNA Mini kit (GC-Biotech, cat#BIO-52073) according to the manufacturer’s instructions. The quality of the obtained RNA was evaluated on the Agilent 2100 Bioanalyzer, and samples with an RNA integrity number (RIN) ≥8.5 were used for gene expression profiling.

For transcriptome profiling, 400 ng RNA was amplified and labelled using 3’ IVT pico kit Affymetrix RNA amplification kit (Nugene) according to the manufacturer’s protocol. Microarray analysis of human small intestinal tissue was performed using Affymetrix Clariom® S PICO HT Plate according to the standard protocols of the Dutch Genomics Service and Support Provider (MAD, Science Park, University of Amsterdam, Netherlands). Data were normalized using Expression Console 1.4.1.46 and microarray results were analyzed using TAC analysis (ThermoFisher Scientific). Data are posted in GEO repository with accession number GSE247523.

For RT-qPCR, 0.5 μg of RNA was transcribed using Revertaid reverse transcriptase (Thermo Fisher Scientific, cat#EP0442). Subsequently, RT-qPCR was performed on a BioRad iCycler (Hercules, CA) using sensifast SYBER No-ROX Kit (GC-biotech, cat#Bio-98020) according to the manufacturer’s protocol. Relative expression levels of genes were calculated in LinRegPCR (version 2015.4, Amsterdam, The Netherlands), and normalized to reference genes. To identify the most stable reference genes, a panel of 12 reference genes was evaluated in GeNorm for various conditions. The list of primer sequences used can be found in the key resources table of this paper.

#### Cytokines stimulation

HFOs donors were stimulated for 72 h with the following cytokines: recombinant human (rh) IFNγ 0,5 ng/ml (R&D systems, cat#285-IF), rhTNFα 2ng/ml (Peprotech, cat#:300-01A), rhIL1β 5 ng/ml (Thermo Fisher Scientific, cat#PHC0814), rhTGFβ 5 ng/ml (Peprotech, cat#:100-21A). Each donor was stimulated on the same day of passaging with either the single cytokines or a combination of the four cytokines renamed cytokine mix. After 72 h, HFOs were collected and processed for RNA isolation or staining. To determine the appropriate concentration per cytokine a 24 h titration was performed by assessing the activation of the downstream target genes with RT-qPCR which for each cytokine mentioned above were respectively: IRF1, NFKBia, IRAK1, PAI (key resources table).

#### Proliferation assay

Cell proliferation was measured using a Click-iT® plus EdU Alexa Fluor® 647 Flow Cytometry Assay Kit (Invitrogen, Thermo Fisher Scientific, cat#C10634). Briefly, 72 h after cytokines stimulation HFOs were incubated with 5-ethynyl-2′-deoxyuridine (EdU) for 4 h. Subsequently cells were harvested in cell recovery solution (Corning®, cat#354253) for 30 mins on ice and single cells were generated by incubating 3D cultures in TrypLE Express (Invitrogen, cat#12605036) for 10 mins at 37°C. Cells were fixated in 4% formaldehyde and permeabilized with saponin, stained for 30 mins at room temperature (RT) protected from light, and acquired by flow cytometry (FACS Diva, BD Bioscience). Results were analyzed using FlowJo software version 10.3 (Treestar, Ashland, OR, USA).

#### Cell death assay

After 72 h stimulation with cytokines, HFOs were harvested in cell recovery solution (Corning®, cat#:354253) for 30 mins on ice and single cells were obtained by dissociation with TrypLE Express (Invitrogen, cat#12605036) for 10 mins at 37°C. Subsequently, cells were stained with Propidium Iodide (Sigma/Aldrich, cat#P4170) 1:1000 and Annexin V (BD Pharmingen BD - cat#556419) 1:33 for 15 mins at RT protected from light and acquired by flow cytometry (FACSDiva, BD Bioscience). Results were analyzed using FlowJo software version 10.3 (Treestar, Ashland, OR, USA).

#### 2D monolayer generation

According to an established protocol^29^ transwell culture inserts (0.4 μm pore size, Cell Quart®, cat#9323012) were coated with 100 μL of 20 μg/mL collagen type I (Ibidi, cat#50201) in 0.01% (v/v) acetic acid (Merk/Millipore, cat#1000631000) for 1 h at RT, and washed with PBS before use. HFOs were expanded and subsequently collected at day 3-5 after passaging. HFOs were incubated with TrypLE Express (Invitrogen, cat#12605036) for 10 mins at 37°C to obtain a single cell suspension. 1∗10^5^ cells/ insert was seeded and maintained in HISC medium containing 10 μM Y-27632 (Sigma, cat#Y0503-5MG), 100 ul in the apical compartment and 600 ul in the basoalteral compartment, for the first 3 days of culture. When a stable monolayer was reached, it was stimulated for 72 h with either single IFNγ (2ng/ml), cytokine mix condition (rhIFNγ 0,5 ng/ml + rhTNFα 2ng/ml + rhIL1β 5 ng/ml + rhTGFβ 5 ng/ml), or control (HISC medium) treatment. After 72 h, the culture medium of the 2D organoid culture was refreshed and the transepithelial electrical resistance (TEER) was measured with a EVOM2 voltohmmeter (World precision instrument, serial#195696), according to the manufacturer’s instruction. Each insert was measured three times (once in each pore) and average values were corrected for background TEER and surface area of the insert to obtain the net-area resistance in Ω∗cm^2^. TEER was measured and the transwell were processed for further assays, fixation, and staining.

#### Enzymatic activity assays

For the enzyme activity assays HFOs were collected from the matrigel in cell recovery solution (Corning®, cat#354253), lysed in 100 ul of cell lysis buffer (Cell signalling/bioke, cat#9803S) and stored -80°C until use. To determine the activity of sucrase-isomaltase and lactase the method developed by Messer and Dahlqvis was used.^55^^,^^56^ Briefly, after a mild sonication on ice for 3 seconds (s), 15 μl of each sample was incubated with 0.01 M sucrose (Sigma-Aldrich, cat#84097-250G) or lactose (Sigma-Aldrich, cat#L3625-1KG) in 0.6 M maleic buffer (Merk/millipore, cat#800380) pH 6.0 for 60 mins at 37°C. To determine the amount of glucose produced by the reaction between the SIS and LCT present in the samples and the respective substrates (sucrose and lactose), 100 ul of the PGO (peroxidase/glucose oxidase, Sigma/Aldrich, cat#P7119-10CAP) / 3,3′-Dimethoxybenzidine dihydrochloride 8mM (Sigma/Aldrich, cat#191248-25G) / 0.5 M Tris–HCl (Sigma/Aldrich, cat#T3253) pH 7 -color solution was added and the absorbance was measured at 450 nm every 5 mins for 30 mins at 37°C. A glucose standard curve was included to determine glucose production. Enzyme activity values were corrected for the total amount of protein, as determined by BCA (bicinchoninic acid) reaction,^57^ and were expressed as μM glucose/μg protein/min. To determine the activity of alkaline phosphatase a diethanolamine assay was used by measuring phosphatase substrate (pNPP), ThermoFisher Scientific, cat#34045) hydrolysis by spectrophotometry according to the manufacturer’s instruction and as previously described.^58^

#### Immunohistochemistry

HFOs and transwell monolayer culture inserts were fixed in 4% formaldehyde for 1 h, encapsulated in HistoGel (Thermo Scientific, cat#HG-4000-012), further embedded in paraffin, and sectioned. Intestinal Alkaline phosphatase (IAP) activity at the brush border was identified with NBT/BCIP conversion (nitro blue tetrazolium chloride/5-bromo-4-chloro-3-indolyl-phosphate, Roche, cat#1681451001) for 15 mins. Images were acquired using an Olympus BX51 microscope and processed with ImageJ (version 1.52a, National Institutes of Health).

#### Transmission electron microscopy

After cytokines stimulation and TEER measurement, transwell membranes were fixed in McDowell fixative containing 4% paraformaldehyde and 1% glutaraldehyde in 0.1 mol/L phosphate buffer for 4 h at RT and later kept at 4°C. Subsequently, samples were postfixed with 1% osmium tetroxide (Electron Microscopy Sciences, Hatfield, PA) in cacodylate buffer, dehydrated in an alcohol series, and embedded in epon (LX-112 resin; Ladd Research, Williston, VT). Subsequently, 80nm epon sections were cut and collected on formvar-coated grids, counterstained with uranyl acetate and lead citrate. Sections were analyzed using a Tecnai-12 G2 Spirit Biotwin electron microscope (Thermo Fisher, Eindhoven, The Netherlands), and images were acquired via a Veleta camera with Radius software (EMSIS, Münster, Germany).

#### Single cell dataset analysis

Publicly available RNAseq dataset (ArrayExpress: E-MTAB-9489) of intestinal fetal cells at 18 weeks GSE^36^ was used to identify the source of IFNγ at the fetal stage.^36^ The Figures shown (Figure S5A) were processed by the Single-Cell module of CLC Genomic Workbench (QIAGEN Digital Insight).

#### Human fetal T cells isolation

After dissociation of the epithelial fraction of human fetal proximal and distal intestinal areas, tissue pieces were minced and digested with collagenase type IV (Sigma, cat#C5138-500MG) 1mg/ml in IMDM medium (Invitrogen, cat#21980-065) for 30 mins at 37°C on a shaker (100 stokes/min).^54^ At the end of the incubation the supernatant was filtered through a 70 μm cell strainer and centrifuged for 10 mins at 500 g 4°C; the pellet was washed once more in PBS for 10 mins at 500 g and 4°C. Subsequently, cells were resuspended in 10 mL HBSS (Westburg/Capricon, cat#HBSS-3A), layered on 4 mL standard isotonic Percoll solution (SIP: 0,24 mL 103 PBS 10x + 2,16 mL Percoll (VWR of Fisher Sc., cat#17-0891-01) + 1,6 mL PBS 1x) and gradient centrifuged for 20 mins at 18°C-22°C at 1000 g (acceleration in 120 sec; 0 break). After the centrifugation, the lymphocyte ring was carefully collected, washed in PBS, and resuspended in 5ml of IMDM medium (Invitrogen, cat#21980-065) supplemented with 1% FBS (Sigma/Aldrich, CAT#F7524) and 1% penicillin/streptomycin (Invitrogen, cat#15140122) and cells were counted with coulter counter machine. T cells were then isolated by positive selection with CD3 MicroBeads (Miltenyi Biotec, cat#130-050-101) according to manufacturer protocol by using MS columns (Miltenyi Biotec, cat#130-042-201). CD3^+^ cell fraction was measured by flow cytometry (Fortessa, BD Bioscience) and quantified using FlowJo software version 10.3 (Treestar, Ashland, OR, USA).

#### T cells/ 3D HFOs co-culture

After enrichment, T cells were counted, and half of the cells (unstimulated T cells - UTC) was plated in Human Intesticult (HI) medium (Stemcell Technologies, cat#06010) around the organoids matrigel-dome (1∗10^5^ cells per organoids well in 500μl HI). The other half of the T cells was activated (stimultated T cells - STC) to produce cytokines by 2 h incubation with phorbol 12-myristate 13- acetate 50 ng/ml (PMA, Santa Cruz/Bio-Connect, cat#sc-3576A) Ionomycin 1000 ng/ml (IONO, Invitrogen, cat#I-24222) at 37°C. Subsequently, cells were counted again and plated in HI medium around the organoids matrigel-dome (1∗10^5^ cells per organoids well in 500μl HI). One well was cultured in absence of T cells in HI medium serving as the control condition. After 72 h incubation, HFOs were harvested in RLY lysis buffer for RNA isolation (ISOLATE II RNA Mini Kit, GC-Biotech, cat#BIO-52073). Both UTC and STC were checked for IFNγ production by enzyme-linked immunosorbent assays (IFNγ DuoSet ELISA, Bio-Techne, cat#DY285B-05).

#### T cells/2D co-culture

After enrichment, CD3^+^ T cells were counted, and half of the cells (UTC) was plated in 600ul HI medium at the basolateral side of the transwell insert (1∗10^5^ cells per transwell). The other half of T cells was activated (STC) to produce cytokines by 2 h incubation with phorbol 12-myristate 13- acetate 50 ng/ml (PMA, Santa Cruz/Bio-Connect, cat#sc-3576A) Ionomycin 1000 ng/ml (IONO, Invitrogen, cat#I-24222) at 37°C. Subsequently, cells were counted again and plated in 600ul HI medium at the basolateral side of the transwell insert (1∗10^5^ cells per transwell). One well was cultured in the absence of T cells in HI medium serving as the control condition. After 72 h co-culture TEER was measured. To validate the effect of IFNγ inhibition on TEER, we performed the same experiment as above by adding IFNγ antibody inhibitor 10μg/ml (R&D, #MAB2851-100) or Mouse IgG2B Isotype Control (R&D, # MAB004, Clone 20116) to each transwell condition for 72 h before TEER measurement.

### Quantification and statistical analysis

For prolonged organoid culture gene expression values were plotted from individual cultures, and changes over time were tested with ratio paired t-test, with p-values < 0.05 considered as statistically significant. To test differences between gestational age groups in fetal tissue gene expression, one-way ANOVA was used, with Tukey’s post-test, and p-values < 0.05 were considered statistically significant. For all other experiments a one-way non-parametric ANOVA was performed with p-values < 0.05 considered as statistically significant.

## Supplemental information

Document S1. Figures S1–S5

Table S1. Differentially expressed genes and comparisons between the developmental fetal and neonatal stages, related to Figure 1 The table is made of 16 sheets named as list 1–16. Each list is divided into 8 columns (A–H). (A) Transcript cluster ID. (B) Bi-weight average signal (log2) intensity first gestational age group. (C) Bi-weight average signal (log2) intensity second gestational age group. (D) Fold change (linear). (E) ANOVA *p* value. (F) False discovery rate (FDR) *p* value. (G) Gene symbol. (H) Gene description. Specific comparisons in each list: 1) 35–36 distal small intestine (SI) vs. 20 weeks distal SI. 2) 35–36 distal SI vs. 18 weeks distal SI. 3) 35–36 distal SI vs. 16 weeks distal SI. 4) 35–36 distal SI vs. 13 weeks distal SI. 5) 20 weeks distal SI vs. 18 weeks distal SI. 6) 20 weeks distal SI vs. 16 weeks distal SI. 7) 20 weeks distal SI vs. 13 weeks distal SI. 8) 18 weeks distal SI vs. 16 weeks distal SI. 9) 18 weeks distal SI vs. 13 weeks distal SI. 10) 16 weeks distal SI vs. 13 weeks distal SI. 11) 20 weeks proximal SI vs. 18 weeks proximal SI. 12) 20 weeks proximal SI vs. 16 weeks proximal SI. 13) 20 weeks proximal SI vs. 13 weeks proximal SI. 14) 18 weeks proximal SI vs. 16 weeks proximal SI. 15) 18 weeks proximal SI vs. 13 weeks proximal SI. 16) 16 weeks proximal SI vs. 13 weeks proximal SI.
