# Supplementary material for: Pro-inflammatory T cells-derived cytokines enhance the maturation of the human fetal intestinal epithelial barrier
Source: iScience. 2024 May 7;27(6):109909. doi: 10.1016/j.isci.2024.109909 (PMC11134877; doi:10.1016/j.isci.2024.109909)
Supplement: Document S1. Figures S1–S5 [file mmc1.pdf]

iScience, Volume *27*

## Supplemental information

# Pro-inflammatory T cells-derived cytokines enhance the maturation of the human fetal intestinal epithelial barrier

**Francesca P. Giugliano, Marit Navis, Sarah Ouahoud, Tânia Martins Garcia, Irini A.M. Kreulen, Evelina Ferrantelli, Sander Meisner, Jacqueline L.M. Vermeulen, Manon van Roest, Jean-Noël Billaud, Jan Koster, Yousif Dawood, Bernadette S. de Bakker, Daisy I. Picavet-Havik, Irene M. Schimmel, Nicole N. van der Wel, Pim J. Koelink, Manon E. Wildenberg, Joep P.M. Derikx, Wouter J. de Jonge, Ingrid B. Renes, Ruurd M. van Elburg, and Vanesa Muncan**

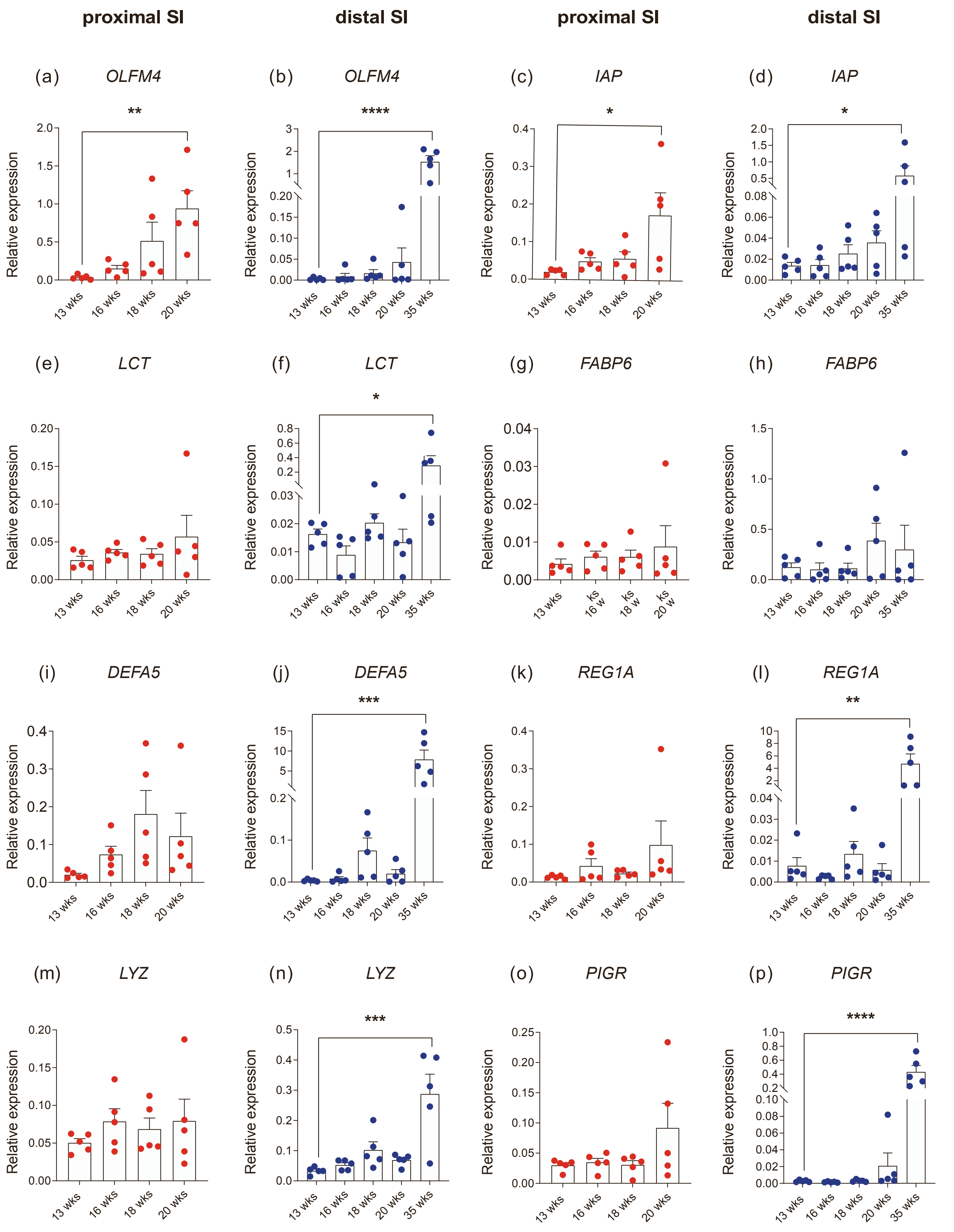


**Figure S1. Gene expression of stem cells, absorption and defense markers is increased in neonatal vs fetal tissue, Related to Figure 1.** RT-qPCR analysis in proximal (red) and distal (blue) human fetal intestinal tissue of **(a-b)** OLFM4, **(c-d)** IAP, **(e-f)** LCT, **(g-h)** FABP6, **(i-j)** DEFA5, **(k-l)** REG1A, **(m-n)** LYZ, **(o-p)** PIGR. n=5 independent Donors per group and data is presented as mean with error bars representing standard error of the mean (SEM). *p<0.05, **p<0.01, ***p<0.001, ****p<0.0001, as determined by non-parametric one-way ANOVA.

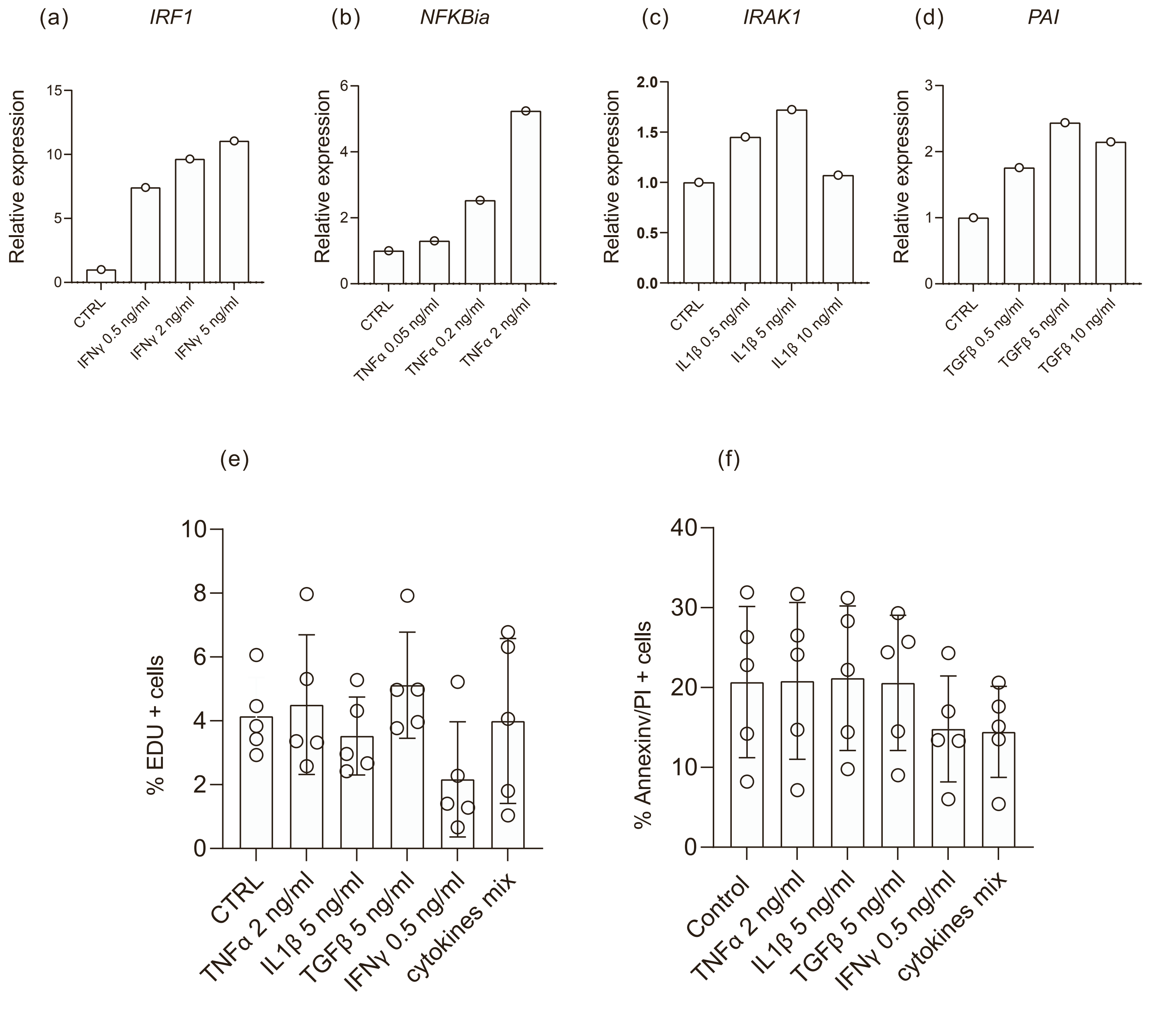


**Figure S2. Gene expression of cytokines downstream targets, cell proliferation and cell death markers in HFOs 24h after cytokines stimulation, Related to Figure 3. (a-d)** RT-qPCR analyses of respectively IFNγ, TNFα, IL1β, and TGFβ downstream target genes **(a)** IRF1, **(b)** NFKBia, **(c)** IRAK1, **(d)** PAI in HFOs. n=1 Donor, data is plotted as paired values of an individual culture (proximal and distal SI were mixed). **(e)** Percentage of proliferating cells measured as EDU incorporation and **(f)** dead cells measured as Annexin5/PI positive cells determined by flow-cytometry. Data were acquired from n=5 independent Donors (proximal and distal small intestine HFOs were mixed) and is plotted as paired values of individual cultures. Data is presented as mean with error bars representing standard error of the mean (SEM).

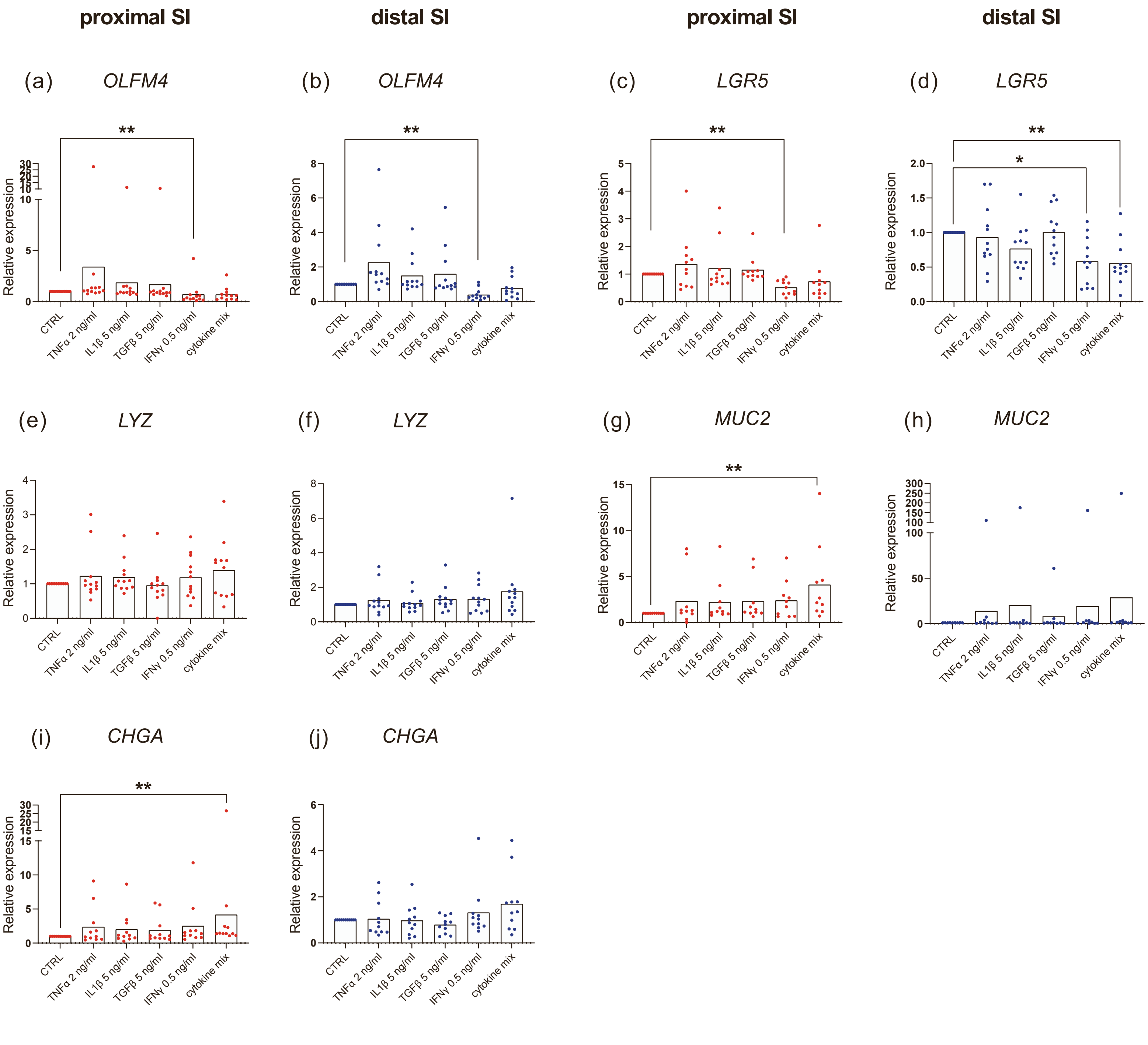


**Figure S3. Stem, Paneth, Goblet and Enteroendocrine cells markers expression upon cytokines stimulation, Related to Figure 3.** RT-qPCR analyses in proximal (red) and distal (blue) small intestine HFOs of **(a-b)** OLFM4, **(c-d)** LGR5, **(e-f)** LYZ, **(g-h)** MUC2 and **(i-j)** CHGA. n=12 independent Donors for OLFM4, LGR5, LYZ, n=11 independent Donors for CHGA, n=10 and 9 independent Donors for MUC2 proximal and distal, respectively. Data is plotted as paired values of individual cultures. *p<0.05, **p<0.01, as determined by non-parametric one-way ANOVA.

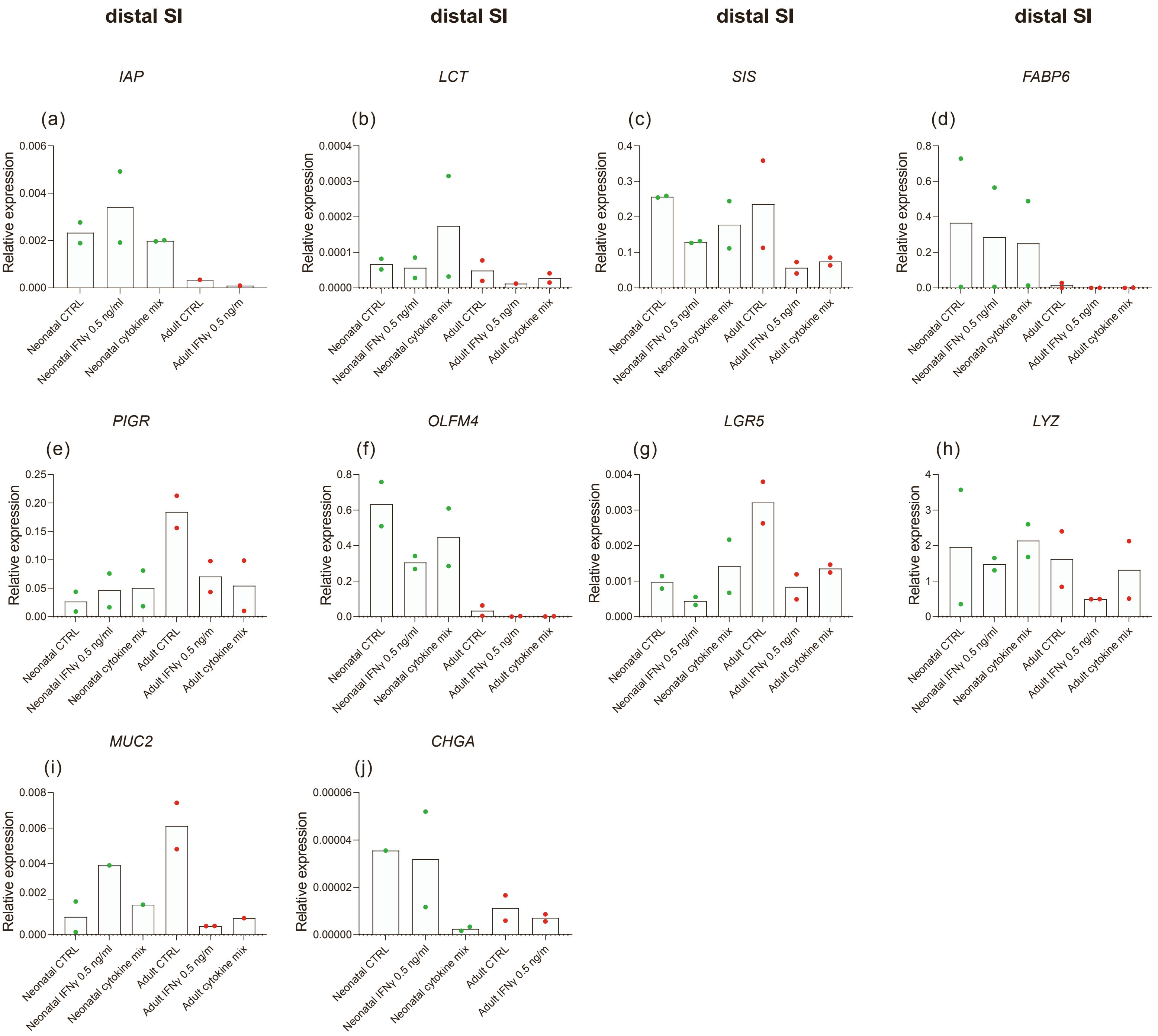


**Figure S4. Brush border, stem, paneth, goblet and enteroendocrine cells markers upon cytokines stimulation in neonatal and adult organoids, Related to Figure 3. (a-j)** RT-qPCR analyses of maturation markers in neonatal (green) and adult (red) distal small intestine organoids of **(a)** IAP, **(b)** LCT, **(c)** SIS, **(d)** FABP6 and **(e)** PIGR, **(f)** OLFM4, **(g)** LGR5, **(h)** LYZ, **(i)** MUC2, **(j)** CHGA. n=2 independent Donors per group, data is plotted as paired values of individual cultures.

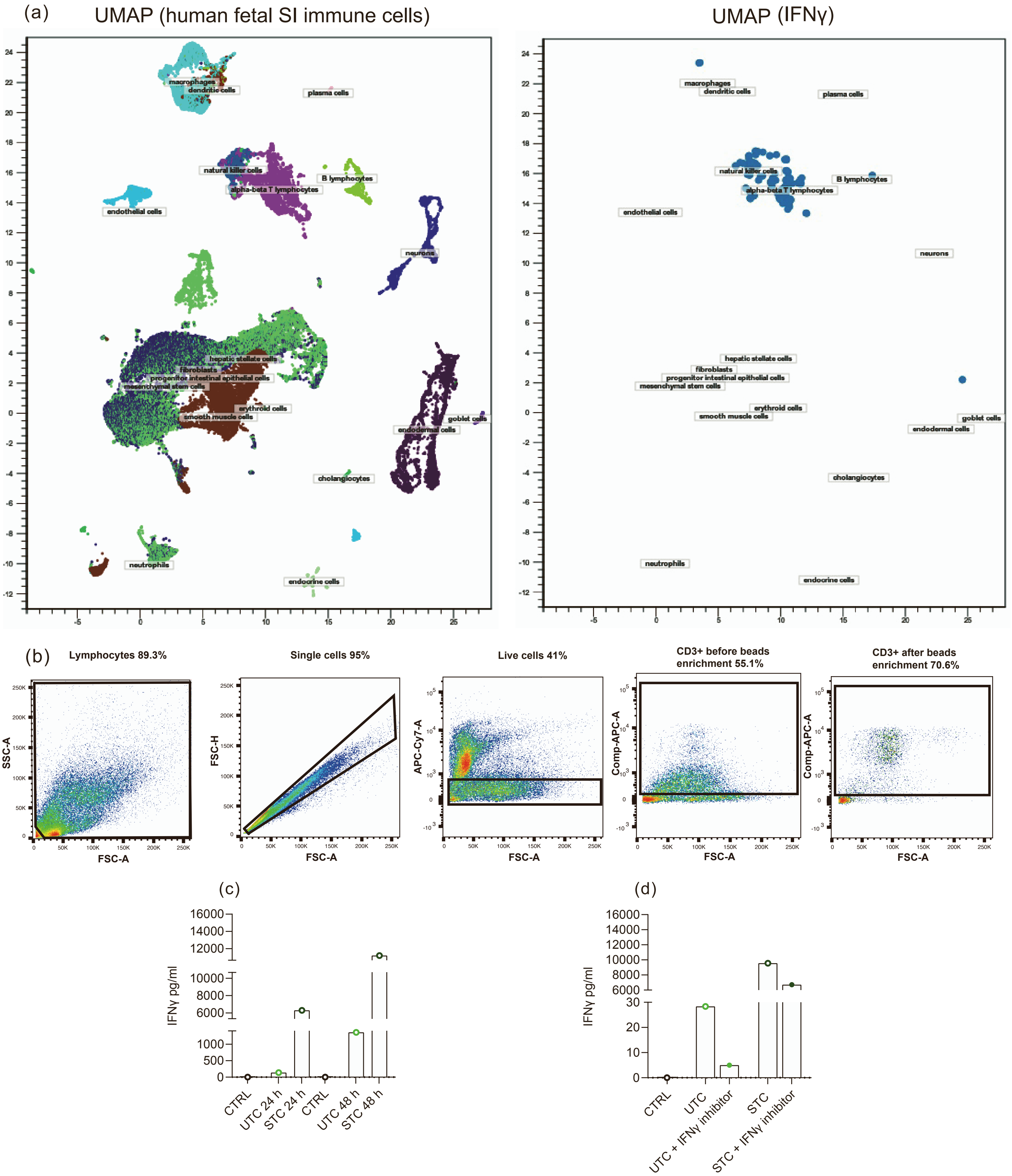


**Figure S5. CD3+ T cells present in human fetal intestinal lamina propria secrete IFNγ both in vivo and in vitro, Related to Figure 6. (a)** UMAP from single cells RNAseq data of fetal intestine shows IFNγ being produced mainly by T cells. **(b)** Flow cytometry analysis of CD3+ T cells sorted with CD3+ beads. **(c)** IFNγ levels measured by ELISA (pg/ml) 24 h after isolation of T cells in both unstimulated (UTC) and stimulated (STC) conditions compared to control (n=1 Donor). **(d)** IFNγ levels measured by ELISA (pg/ml) with or without the presence of IFNγ inhibitor (n=1 Donor).
